# Saltwater icephobicity: Influence of surface chemistry on saltwater icing

**DOI:** 10.1038/srep17563

**Published:** 2015-12-02

**Authors:** Katherine Carpenter, Vaibhav Bahadur

**Affiliations:** 1 Department of Mechanical Engineering, Texas Materials Institute, University of Texas at Austin, Austin, TX 78712.

## Abstract

Most studies on icephobicity focus on ice formation with pure water. This manuscript presents studies to understand the influence of surfaces on saltwater ice nucleation and propagation. Experiments are conducted to quantify the influence of surface chemistry on saltwater ice nucleation and to understand the utility of superhydrophobic surfaces for saltwater icephobicity. These experiments are conducted with pure water and two sodium chloride solutions, which represent the salinity of seawater and briny produced water. It is seen that the presence of salt slows down the ice front propagation velocity significantly. Saltwater droplet impact dynamics on superhydrophobic surfaces are also different from pure water. Saltwater droplets retract more and a greater fraction of impacting liquid is repelled from the superhydrophobic surface. It is seen that the greater bounciness of saltwater droplets is a result of slower ice nucleation propagation kinetics. These experiments indicate that superhydrophobic surfaces will have better resistance to impact icing with saltwater than pure water and can remain useful at temperatures as low as −40 °C. Overall, this work is a starting point for further studies on heterogeneous nucleation in saltwater and serves as a bridge between the widely studied freshwater icephobic surfaces and saltwater-related applications.

The past two decades have seen significant research on icephobicity with the objective of developing surfaces to prevent or reduce icing. Icephobicity-related research can be broadly categorized as studies on heterogeneous nucleation^1^,^2^, ice adhesion^3^,^4^,^5^, droplet impact^6^,^7^,^8^,^9^, and applied research on active and passive methods for deicing and anti-icing. Overall, ice formation is a complex phenomenon with surface chemistry/wettability^10^,^11^,^12^,^13^,^14^, surface elasticity^6^, and temperature/humidity^15^ affecting icing. Low-energy surfaces can lessen ice buildup via reduced ice-surface adhesion and/or delay in ice nucleation on such surfaces. The fundamental science has been translated into commercial icephobic coatings for applications in infrastructure, aerospace, and energy.

An overwhelming fraction of existing literature studies icephobicity of pure water. There is very little research on saltwater ice mitigation and heterogeneous freezing in saltwater, which is the focus of the present work. While the well-known thermodynamic freezing point depression by salt addition does reduce the probability of ice formation, ice will nevertheless form at low enough temperatures. The role of surface chemistry on heterogeneous nucleation in saltwater has not been examined to date. The utility of superhydrophobic surfaces for marine (saltwater) icephobic applications is largely unknown.

Saltwater ice mitigation has many important applications. Saltwater ice formation on offshore oil-gas platforms, ships, and other marine structures can cause structural and safety problems^16^. Ice management strategies do exist for marine icing, including chemicals, coatings, structure designs, heating, and manual deicing^16^. However, these deicing/anti-icing technologies are based on freshwater icing studies without much analysis on the unique aspects of saltwater ice. Another application where an understanding of saltwater freezing is critical is desalination via freezing^17^,^18^,^19^. The kinetics of freezing of salt solutions is not well characterized, and there are very few experimental studies on this topic.

A synopsis of the limited research on saltwater icing is presented below. Many studies have focused on the freezing point depression by salt addition^20^. Khvorostyanov and Curry^21^ developed analytical expressions for freezing temperatures as a function of water composition, solute concentration, and ambient pressure. Experimental work by Pruppacher^22^ demonstrated that salt addition affects the ice crystal structure and that the growth rate depends on the type of salt and concentration. Ayel *et al.*^23^ investigated the crystallization kinetics of water-MPG (monopropylene glycol, commonly used anti-freeze) mixtures and showed that growth rate increases with supercooling and decreases with increasing MPG mass fraction. This is due to slower migration of water molecules in the presence of anti-freeze and increased anti-freeze concentration in the remaining liquid, which reduces the equilibrium temperature. Bauerecker *et al.*^24^ imaged the freezing of saltwater droplets under homogeneous freezing conditions and used a molecular dynamics (MD) simulation to gain insights into experimental results. The observed decrease in the freezing propagation rate with an increase in salt concentration is due to the additional time required to reject salt ions into the unfrozen volume. This slow-down in freezing propagation has also been reported from other MD simulations^25^,^26^.

It should be noted that the above saltwater-related studies focus on ice nucleation and propagation without trying to uncover the role of the surface. Based on freshwater icephobicity studies, it is expected that surface texture and chemistry will strongly influence heterogeneous saltwater icing. This has been confirmed by very limited studies, such as Zobrist *et al.*^27^ and Wilson and Haymet^28^, which investigated heterogeneous nucleation with different kinds of ice nuclei (nonadecanol, silica, silver iodide, Arizona dust, and sand grains) inside the liquid.

To summarize, there are no comprehensive studies on heterogeneous nucleation in saltwater, or on saltwater ice mitigation. The present work fills this huge and important gap in the field of icephobicity. We present experimental studies of saltwater freezing under static (stagnant water) and dynamic (liquid impact) conditions. We isolate the influence of surface chemistry on the heterogeneous nucleation kinetics of saltwater solutions by quantifying the ice nucleation temperatures and ice front propagation kinetics. These statistically significant experiments are conducted with three different solutions: pure water, a 0.6 molar sodium chloride (NaCl) solution, and a 3 molar NaCl solution. The 0.6 M solution is representative of the average salinity of seawater, and the 3 M solution represents very briny produced water. For reasons of brevity, the 0.6 M and the 3 M solutions are referred to as seawater and brine, respectively, in the rest of this manuscript. After studying static freezing of saltwater solutions, we analyze impact dynamics of the three liquids on superhydrophobic surfaces. We define a superhydrophobic surface as one that can completely repel water droplets under room temperature conditions. These experiments uncover the role of salt concentration on impact dynamics and quantify the effectiveness of superhydrophobic surfaces in repelling saltwater ice. It is again noted that all the results in this work are baselined against similar experiments with pure water to highlight the differences.

## Results

### Ice nucleation in static saltwater droplets

Several studies have characterized freezing kinetics in terms of the induction time^10^,^29^,^30^. Alternatively, other studies have characterized freezing kinetics in terms of the freezing temperature as the surface is cooled down^1^,^13^,^31^,^32^,^33^. While both the induction time and freezing temperature can be used to characterize the influence of a surface on nucleation, this study uses the latter approach.

**Figure 1:**
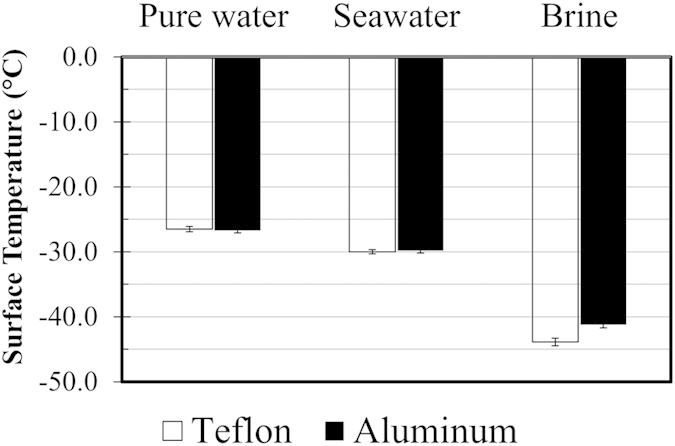
Average freezing temperature of pure water, seawater, and brine on Teflon and aluminum surfaces. Each bar represents the average of 200 freezing instances.

Figure 1 shows the average measured freezing temperatures for pure water, seawater, and brine droplets on aluminum and Teflon surfaces (see Methods section for details of the experiments and surfaces). Each bar in Fig. 1 is the average of two hundred measurements over four sets of experiments involving fifty droplets each. It should be noted that the temperatures in Fig. 1 are the surface-water interface temperatures, which are calculated based on the plate temperature and the temperature at the top of the droplet^29^. The error bars were calculated using the t-value and a 95% confidence interval, defined as 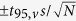, where *t*_*95,ν*_is the t-value for the 95% confidence interval, *N* is the number of samples, *s* is the standard deviation of the *N* samples, and *ν* is the degrees of freedom, which is *N-1*.

Figure 1 shows the freezing point depression as the salt concentration increases. It is seen that brine droplets freeze at least 15 °C lower than pure water and were observed to remain liquid at temperatures approaching −45 °C. Interestingly, surface chemistry does not have a measureable influence on the heterogeneous freezing temperatures of pure water and seawater, i.e., the average freezing temperatures were similar on both the Teflon and aluminum surfaces. However, there is a difference in the freezing temperature of brine droplets on Teflon versus aluminum surfaces, with a lower freezing temperature on the Teflon surface.

These experiments provide insights into the mechanisms involved in heterogeneous freezing of saltwater solutions. Firstly, it was verified via high-speed imaging that nucleation originated at the three-phase contact line for pure water and saltwater. These results imply that surface chemistry on its own does not affect nucleation initiation; the differences in the freezing temperatures can be attributed to the thermodynamic freezing point depression with salt addition. There is a 3 °C difference in the nucleation temperatures of brine droplets on Teflon and aluminum. While the reasons for this difference are not clear, one possible explanation is frost formation near the droplet, which is difficult to prevent at such low temperatures. Frost formation rates were observed to be higher on aluminum than Teflon, and frost formation could have triggered ice formation in droplets on the aluminum surface at higher temperatures. Overall, the present statistically relevant experiments show that surface chemistry on its own does not substantially alter the freezing temperature of static saltwater and pure water droplets. This also implies that any enhanced icephobicity benefits of superhydrophobic surfaces with saltwater can be attributed to surface roughness effects and the difference in impact characteristics of pure water and saltwater droplets.

### Saltwater droplet impact dynamics

**Figure 2:**
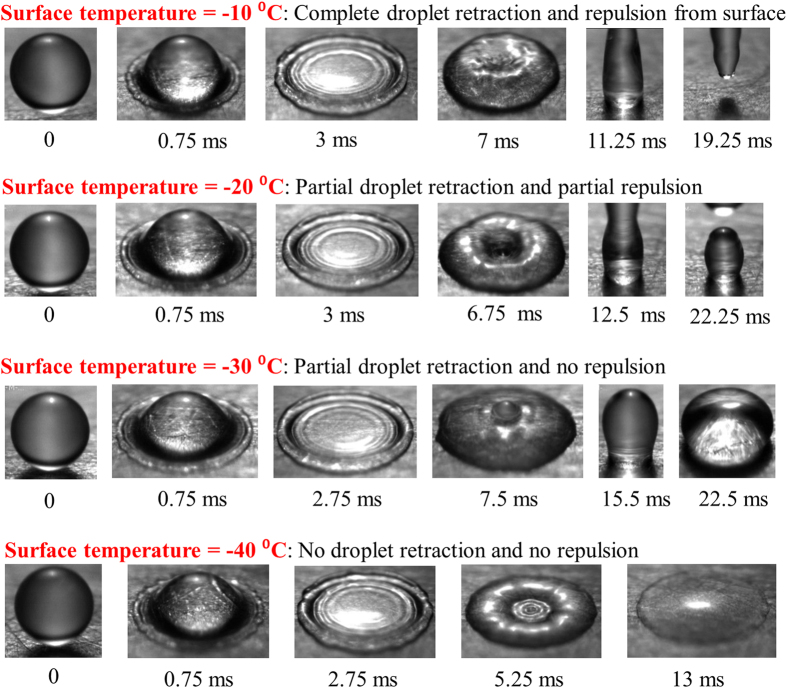
Contact line retraction and repulsion of pure water droplets (2.2 mm initial diameter) on a superhydrophobic surface at (**a**) −10 °C, (**b**) −20 °C, (**c**) −30 °C and (**d**) −40 °C.

Droplet impact dynamics was experimentally studied to estimate the differences in saltwater versus pure water dynamics, and judge the utility of superhydrophobic surfaces in preventing saltwater ice. Figure 2 shows images of pure water droplets impacting the superhydrophobic surface (fabrication details in Methods section) at surface temperatures of −10 °C, −20 °C, −30 °C and −40 °C. In all these experiments, the spreading stage lasted for 2–3 ms, where the droplet spread to approximately 6 mm. The retraction profiles, however, vary significantly, and the extent of retraction depends on the surface temperature. At −10 °C, the water droplet retracts completely under the action of surface tension and bounces off the surface. At −20 °C, the droplet does not completely retract, and the entire droplet volume is not repelled from the surface. At −30 °C, droplet retraction is further subdued. This behavior has been documented by other researchers^9^,^34^, who report the transition temperature at which a droplet fails to bounce off a superhydrophobic surface as between −20 °C to −30 °C, which matches the present observations. A drastic change is seen at −40 °C, wherein water droplets do not retract at all and remain pinned in the maximum spreading position. All these differences are seen in Video 1 in the supplementary information section.

**Figure 3:**
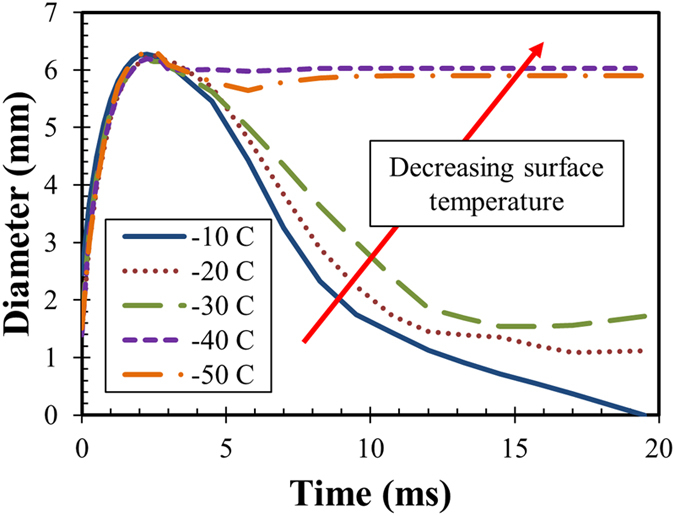
Time-dependent position of the contact line for pure water droplet impact at different surface temperatures.

Image processing can provide quantitative data for use in analyses and comparisons. Figure 3 shows the time-dependent droplet diameter during spreading and retraction at various surface temperatures with pure water droplets. The spreading phase is the same and independent of surface temperatures. However, the retraction profiles are markedly different, as depicted in Fig. 3. In general, lower temperatures make retraction more sluggish, which matches the observations of Alizadeh *et al.*^12^.

**Figure 4:**
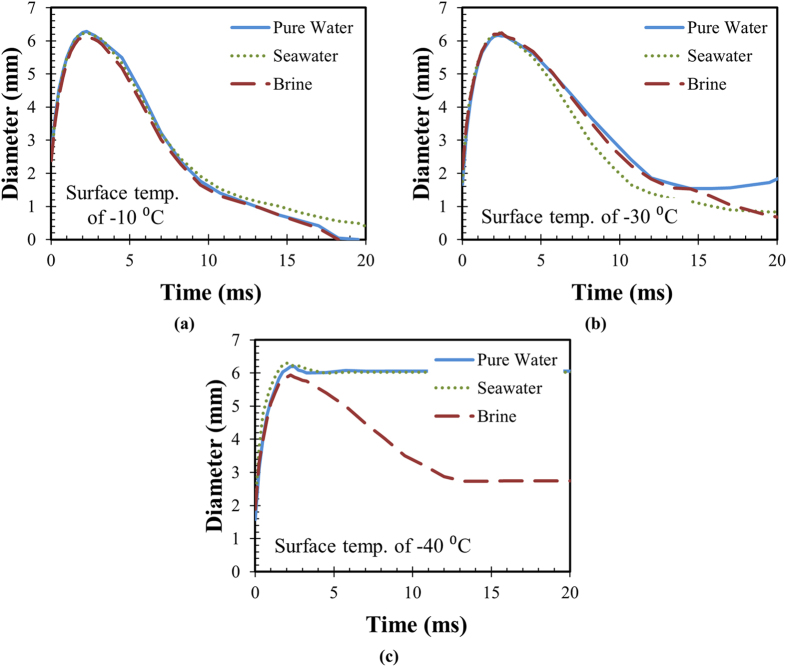
Time-dependent droplet diameter for the three liquids at surface temperatures of (**a**) − 10 °C, where all droplets are repelled, (**b**) −30 °C, where no droplet is completely repelled, but the retraction characteristics are markedly different, and (**c**) −40 °C, where only brine droplets show retraction.

An important objective of this work is a comparison of saltwater droplet impact dynamics at various surface temperatures. Figure 4a shows the impact dynamics of the three liquids at −10 °C. It is seen that all three liquids show identical profiles during the spreading stage. The retraction profiles vary slightly, but all three droplets have enough energy to completely come off the surface. Figure 4b shows the impact dynamics at −30 °C for the three liquids. It is seen that the retraction profiles are noticeably different with the brine droplet retracting more than the other two droplets. While none of the droplets completely leave the surface, the brine and saltwater droplets recoil significantly more than pure water droplets. Figure 4c compares the impact behavior at −40 °C for the three types of fluids. Both pure water and seawater show poor retraction, but the brine droplet is able to retract about halfway. Differences in the retraction profiles are evident in Video 1 in the supplementary information section.

Based on analyses of droplet impact patterns, we propose two parameters to characterize the effectiveness of a surface in reducing ice buildup upon liquid impact. The first parameter is the retraction ratio after droplet impact, with the final retraction position normalized by the maximum spreading diameter. A value of 1 corresponds to complete expulsion of the droplet from the surface, which implies icephobicity under liquid impact conditions. However, this parameter on its own cannot completely quantify the anti-icing performance of a surface. In many experiments, it was observed that the droplet does not retract completely, but significant volumes of the droplet pinch out from the pool of liquid stuck to the surface. The second parameter to judge the effectiveness of a superhydrophobic surface is the fraction of the liquid repelled after impact. This parameter was measured by estimating the volume of liquid left behind on the surface via image processing. A value of 1 implies that no fraction of the droplet is left behind, which translates to complete icephobicity upon liquid impact. It is important to note that conclusions about the effectiveness of superhydrophobic surfaces in repelling water are made by jointly considering both these parameters and not by considering a single parameter alone. It should also be noted that a retraction ratio of 1 implies that the fraction of the liquid repelled is also 1 by definition. However, the reverse is not true in cases where liquid is ejected off a droplet that has stopped retracting.

**Figure 5:**
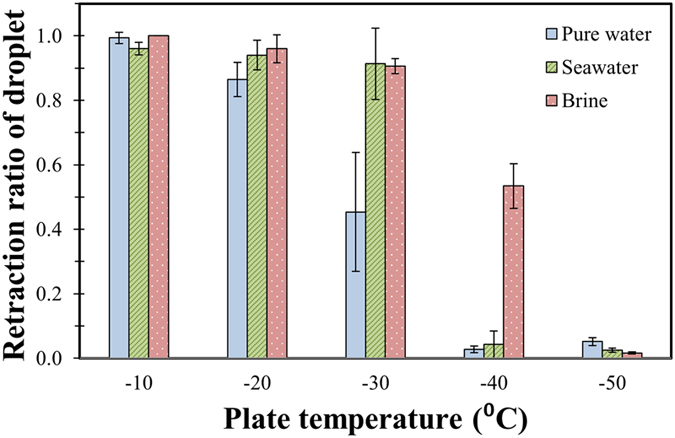
Retraction ratio after droplet impact of three liquids on a superhydrophobic surface at various temperatures.

**Figure 6:**
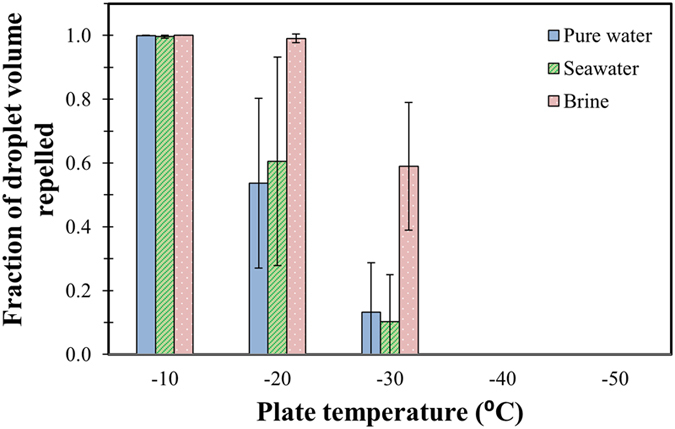
Fraction of liquid repelled after droplet impact of three liquids on a superhydrophobic surface at various temperatures.

Figures 5 and 6 show the two parameters (retraction ratio and fraction of liquid repelled) for the three liquids at various surface temperatures. It should be noted that these results are the average of at least five experiments; the error bars are calculated using the t-value and a 95% confidence interval. As can be clearly observed from the two plots, all three fluids transition from complete retraction and bouncing off the surface at −10 °C to no retraction (Fig. 5) and no liquid repelled at −50 °C (Fig. 6). We define the transition temperature range as one over which the retraction ratio changes significantly. Importantly, this transition temperature depends on the salt concentration when other parameters are held constant. At −20 °C, all three solutions show very high droplet retraction (Fig. 5); however, the pure water and the seawater droplets have substantial liquid left on the surface, whereas the brine droplet is completely repelled (Fig. 6). At −30 °C, much more liquid repulsion is observed for brine droplets (Fig. 6); this corresponds to the higher retraction of seawater and brine droplets as compared to pure water (Fig. 5). Also, at  30 °C, the retraction ratio of pure water droplets decreases substantially when compared to −20 °C. At −40 °C, all the three solutions show zero liquid repulsion upon droplet impact (Fig. 6); however, the retraction profiles (Fig. 5) are different, with brine droplets retracting the most. Additionally, at −40 °C, the retraction ratio of the seawater droplets decreased significantly compared to −30 °C. For brine droplets, the retraction ratio decrease was not as large as that of the seawater droplets.

Figures 5 and 6 can be used to make conclusions regarding the utility of superhydrophobic surfaces for saltwater icephobicity applications. It is seen that superhydrophobic surfaces will display saltwater repellency at lower temperatures than those with pure water. While superhydrophobic surfaces are unable^9^ to repel pure water at temperatures below −20 °C, they can still repel saltwater at temperatures below −30 °C, i.e., both the retraction ratio and fraction of droplet volume repelled is greater for saltwater and brine droplets. This increases the useful working temperature range of superhydrophobic surfaces in marine conditions. The addition of salt reduces the sluggishness in droplet retraction at lower temperatures. Higher retraction with salt water or brine will thus reduce the contact area and decrease the ice-surface adhesion for ice removal applications. Overall, it can be concluded that superhydrophobic surfaces will show moderately improved performance at preventing saltwater from freezing upon impact compared to pure water.

## Discussions

This section analyzes the observed differences in the impact dynamics of saltwater droplets. The ability of a droplet to successfully come off the surface after impact depends on the rate at which energy is lost during droplet spreading and retraction. Sources of droplet energy loss include viscous losses at the fluid-solid interface^11^,^35^, contact line friction, and losses due to substrate deformation^6^. These effects are analyzed to estimate their contribution in explaining the differences in the icephobicity of saltwater droplets.

Estimating the viscous losses during droplet impact is not straightforward due to the complex fluid mechanics involved. To a first order, the viscous loss during droplet impact can be approximated as^6^,^35^:


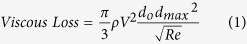


where *ρ* is the density, *V* is the impact velocity, *d*_*o*_ is the initial diameter, *d*_*max*_ is the maximum diameter, and *Re* is the Reynolds number, defined as 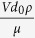, where *μ* is the dynamic viscosity. The viscous loss scales as (*ρμ*)^1/2^ for droplets with the same diameter and impact velocity. However, the viscosity and density for saltwater are not very different from pure water^36^ (details in the supplementary information section). Even though viscous losses increase at lower temperatures, this cannot completely explain the differences in the retraction between saltwater and pure water droplets. This observation is further confirmed by estimating the fraction of the droplet which sees a viscosity change with temperature. The thermal penetration depth is a measure of the distance in the liquid that the effect of the cold surface is felt. Since viscosity increases at lower temperatures, a larger penetration depth will translate to larger viscous losses and more rapid energy loss. The thermal penetration depth scales as 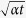, where 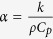 is the thermal diffusivi*t*y, *t* is time, *k* is the thermal conductivity, *ρ* is density, and *C*_*p*_ is the specific heat. However, the estimated thermal penetration depths are approximately the same for all three fluids in this study as the properties^32^ do not change significantly (more details in the supplementary information section).

Based on the above analyses, it is unlikely that differences in thermophysical properties are the primary reason for the difference in the impact dynamics between saltwater and pure water droplets. A more likely explanation is based on the differences in the probabilities of ice nucleation during the spreading and retraction stages. Figure 5a shows that all three solutions have similar retraction at −10 °C; differences emerge at lower temperatures as the probability of icing increases. Clearly, pure water droplets will have a higher tendency to nucleate ice than saltwater droplets; however, ice formation and propagation will be limited in the ~10 ms timescale associated with droplet impact. An important consideration is the two-stage nature^24^ of ice formation. Ice formation is typically broken into two stages, consisting of an initial superfast stage, followed by a much slower second stage. In the first freezing stage^24^, the entire liquid mass becomes cloudy and establishes the scaffolding on which the remainder of the water solidifies. The second stage is much slower and involves propagation of the freezing front from the bottom of the droplet to the top. These two stages of freezing are clearly seen in Video 2 in the supplementary information section.

The differences in the impact dynamics of saltwater and pure water are attributed to the differences in the propagation rates of the initial stage. It is known that the presence of salt and impurities slows down the initial freezing stage because the impurities must be excluded from the ‘frozen’ crystal structure^24^. This implies that the pure water droplet sees a much larger penetration of the first freezing stage than saltwater droplets during the time that the droplet contacts the surface. The initial freezing stage affects the interfacial and liquid properties since the liquid changes to a semi-solid mushy state. The resulting increase in the resistance to fluid motion hinders the retraction of pure water droplets, which can explain the differences with saltwater droplets.

**Figure 7:**
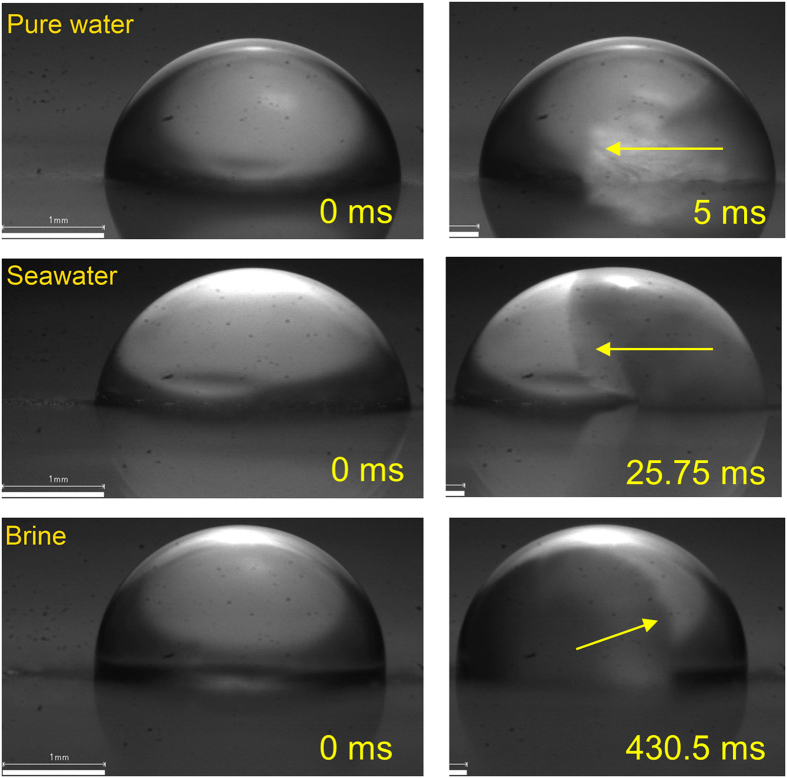
Stage one freeze propagation in pure water, seawater, and brine. The arrows indicate the edge and direction of the moving front. The freeze front propagation velocity reduces with an increase in salt concentration.

This hypothesis was verified by measuring propagation velocities of the initial freezing stage in static pure water and saltwater droplets. High-speed imaging at 4000 fps was employed for these experiments. Figure 7 shows images of the three droplets at the time instant corresponding to approximately half way of the first freezing stage. Table 1 shows the average freeze times and the propagation velocities for the three liquids. It is seen that the propagation velocities in saltwater are much lower than pure water; furthermore, the propagation velocities decrease nonlinearly with the salt concentration. The initial freezing stage is slower in brine by 140× compared to pure water. This implies that most of the brine droplet will not be affected by the initial freezing stage during its residence time on the surface; this reduces energy loss in the brine droplet, allowing greater retraction. On the other hand, the timescale for the initial freezing stage in pure water is comparable to the droplet residence time; this implies that there is significant freezing (first stage) in the pure water droplet during retraction, which explains its sluggishness. Video 3 in the supplementary information section shows the initial freezing stage of all three fluids. It is noted that high-speed visualization to detect freezing is not possible under droplet impact conditions. The above hypothesis represents a plausible mechanism for the differences in the retraction characteristics of different fluids. Other effects, such as viscous losses and contact line friction, will also influence droplet impact dynamics of saltwater solutions. Overall, the lower freezing probability (due to lower freezing temperatures) and slower freeze propagation rates make saltwater droplets bouncier than pure water droplets.

To conclude, this study presents a fundamental investigation of the differences in the freezing characteristics and impact dynamics of saltwater droplets. It is seen that surface chemistry does not directly affect the freezing temperature of static saltwater droplets. The impact dynamics of saltwater droplets are also markedly different from pure water droplets; saltwater droplets demonstrate more retraction and repulsion from the surface. These results imply that existing superhydrophobic surfaces will be more effective at preventing saltwater impact icing than freshwater impact icing. This study also highlights the significant variations in the freezing rate of various saltwater solutions; knowledge of the freeze propagation rates is of critical importance in the development of applications, such as freezing desalination. While the present results are based on single droplet studies, parameters such as droplet size and droplet-droplet interaction are also important considerations. Such parametric variations can be the focus of follow-up studies; furthermore, the trends reported in this manuscript are expected to remain valid.

## Methods

Two types of experiments were conducted to isolate the differences in the freezing characteristics of saltwater versus pure water and are described ahead.

### Experiments on ice nucleation in static saltwater droplets

The first set of experiments measured the ice nucleation temperature and the freezing propagation kinetics of static droplets of three fluids on two different surfaces. The first solution was deionized (DI) water, henceforth referred to as pure water. The other two solutions were 0.6 M NaCl (seawater) and 3 M NaCl (brine). Freezing characteristics were recorded for two hundred droplets for every liquid-surface combination; the present experiments thus represent statistically meaningful measurements unlike many previous studies. To establish the role of surface chemistry on nucleation kinetics, experiments were conducted on two surfaces with contrasting chemistry. Hydrophilic aluminum surfaces (high surface energy^37^ of 169 mJ/m^2^) and hydrophobic Teflon-coated surfaces (low surface energy^37^ of 19 mJ/m^2^) were used in this study.

**Figure 8:**
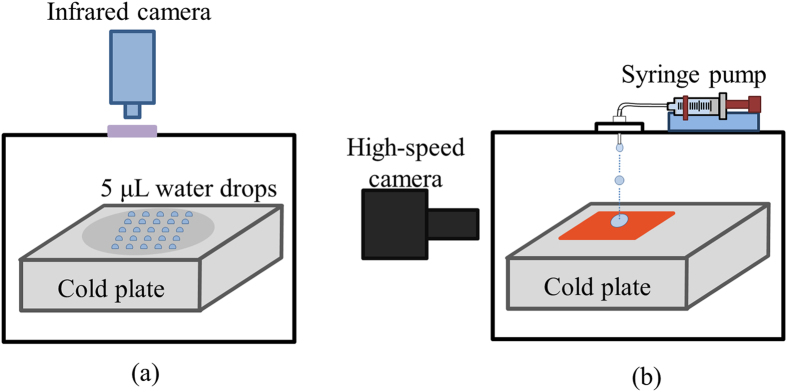
Schematic of the experimental setup for (**a**) studying nucleation characteristics of static saltwater droplets, and (**b**) saltwater droplet impact on superhydrophobic surfaces.

Figure 8 shows a schematic of the experimental setup. All experiments were conducted in a custom-built, air-tight acrylic environmental chamber. The experiments were conducted on a 15 cm by 15 cm plate, which was cooled by liquid nitrogen. To eliminate frost formation, the chamber was flushed with nitrogen. The chamber had an IR transparent germanium window for measuring temperatures at the top of the droplets. Additional details of the setup are provided in the supplementary information section.

The experiments measured the freezing temperature and freeze front propagation kinetics in 5 μL droplets. Initially, the plate temperature was set at 15 °C, and multiple droplets were deposited on the surface. The chamber was then closed and flushed with nitrogen to drive out moisture. When the relative humidity reached less than 1%, the infrared (IR) camera was turned on, and the plate was set to cool at 5 °C/min. The experiment ended when all droplets had frozen, which could be visually observed by the droplets going from clear to opaque.

The temperature at which freezing is initiated was obtained by examining the temperature-time cooling plot of individual droplets as recorded by the IR camera. At the onset of freezing, there is a sudden release of the latent heat of freezing; this heat release occurs very rapidly (in a few microseconds)^12^. The nucleation temperature can be estimated by tracking the thermal signature of the droplet; upon nucleation temperature, a sudden temperature spike is seen. It should be noted that this technique has been previously used by multiple researchers to detect the onset of freezing^12^,^24^,^38^.

We would like to clearly highlight some important aspects of these experiments. Firstly, the reported nucleation temperatures are averages of a very large data set. Two hundred droplets were evaluated for a particular surface-fluid combination. Such statistically relevant data is not reported in most nucleation studies. Secondly, great care was exercised to ensure that salt contamination did not affect the measurements. To reduce the possibility of contamination, each surface was thoroughly cleaned after each experiment (acetone, isopropanol, DI water) and then dried on a hot plate. Thirdly, since the experiments are targeted at isolating the influence of surface chemistry on nucleation, the entire setup was mounted on a vibration-free table. Common external triggers for nucleation, such as shocks and air drafts, are substantially eliminated in this setup.

### Experiments on saltwater droplet impact

The second set of experiments was conducted with the objective of assessing the utility of superhydrophobic surfaces for saltwater ice mitigation. The primary avenues to reduce ice buildup include nucleation delay and reduced ice adhesion. It is expected that the benefits of superhydrophobic surfaces will be amplified by the intrinsic freezing point depression of saltwater. The impact icephobicity of a superhydrophobic surface can be characterized^9^ by the ability of the surface to repel water at low temperatures. Mishchenko *et al.*^9^ estimated the ice prevention ability of superhydrophobic surfaces by studying single droplet impact dynamics to estimate the lowest temperature at which droplets are repelled. Multiple studies^9^,^30^ have discovered that superhydrophobic surfaces repel pure water at temperatures as low as −20 °C to −30 °C; it is expected that saltwater will be repelled at even lower temperatures. This hypothesis was the basis of the present impact experiments, in which the ability of a superhydrophobic surface to repel saltwater at low temperatures was investigated.

The droplet impact experiments (Fig. 1b) were conducted in the same environmental chamber as the static tests. 2.2-mm diameter droplets of the three liquids (pure water, seawater, and brine), at room temperature were released from a syringe pump from a height of 20 cm, which corresponds to an impact velocity of 2 m/s on the cold plate. Droplet impact dynamics was recorded using a high-speed camera at 4000 fps. Before each test, the chamber was flushed with nitrogen, and the plate was cooled to the desired temperature. Five minutes later, a droplet was released to impact the surface, and the droplet-surface interaction was recorded by a high-speed camera. After each impact experiment, the surface was moved, and the next impact experiment was conducted without opening the chamber. This procedure was repeated for all three liquids, and at least five droplet impact experiments were conducted per surface per temperature for each liquid. Note that the surface temperature is essentially the same as the plate temperature. More details on surface temperature estimation are provided in the supplementary information section.

The superhydrophobic surface used in these experiments was a roughened copper surface with a ~1 μm thick spincoated Teflon layer. The static contact angle of a pure water droplet on this surface was ~131°. At room temperature, a 2.2-mm pure water droplet could bounce approximately 4-6 mm off this surface. Fabrication details and surface metrology measurements are provided in the supplementary information section.

It should be noted that great care was taken to avoid surface contamination in these experiments. In particular, each impact experiment was conducted on a different portion of the substrate; numerous samples were fabricated in view of the large number of experiments. These surfaces had consistent surface chemistry and structure, which was verified by surface roughness and contact angle measurements. Details of the experimental setup, sample preparation, measurements, calculations, and data analysis are provided in the supplementary information section. This section also describes the method to detect the onset of freezing. Tabulated thermophysical properties of the three fluids considered in this study are provided. Additionally, the following videos are included: 1) impact dynamics of pure water, seawater, and brine droplets on a superhydrophobic surface at temperatures ranging from -10 °C to -50 °C, 2) illustration of the initial freezing stage and second freezing stage in a pure water droplet, and 3) initial freezing stage propagation in pure water, seawater, and brine droplets.

## Additional Information

**How to cite this article**: Carpenter, K. and Bahadur, V. Saltwater icephobicity: Influence of surface chemistry on saltwater icing. *Sci. Rep.* **5**, 17563; doi: 10.1038/srep17563 (2015).

## Supplementary Material

Supplementary Video 1

Supplementary Video 2

Supplementary Video 3

Supplementary Information

## Figures and Tables

**Table 1:** Duration of initial freezing stage and propagation velocity during the initial freezing stage in three liquids.

	Duration of initial freezing stage (ms)	Propagation velocity during initial freezing stage (mm/ms)
Pure water	12.7	0.28
Seawater (0.6 M NaCl)	69.4	0.045
Brine (3 M NaCl)	1720	0.002
