# Supplementary material for: Saltwater icephobicity: Influence of surface chemistry on saltwater icing
Source: Sci Rep. 2015 Dec 2;5:17563. doi: 10.1038/srep17563 (PMC4667180; doi:10.1038/srep17563)
Supplement: Supplementary Information [file srep17563-s4.pdf]

Supplementary Information

Saltwater icephobicity: Influence of surface chemistry on saltwater icing

*Katherine Carpenter, Vaibhav Bahadur**

Department of Mechanical Engineering,

Texas Materials Institute,

University of Texas at Austin, Austin, TX 78712

*Corresponding author: vb@austin.utexas.edu

Details of the experimental setup, sample preparation, measurements, calculations, and data analysis are provided in this section. This section also describes the method to detect the onset of freezing. Tabulated thermophysical properties of the three fluids considered in this study are provided. Additionally, the following videos are included: 1) impact dynamics of pure water, seawater, and brine droplets on a superhydrophobic surface at temperatures ranging from -10 ^{o}C to -50 ^{o}C, 2) illustration of the initial freezing stage and second freezing stage in a pure water droplet, and 3) initial freezing stage propagation in pure water, seawater, and brine droplets.

Details of experimental setup

The germanium IR window was purchased from ISP Optics and had an average transmittance greater than 0.96 in the 7-13 μm wavelength range. The cold plate was purchased from Instec. The IR camera was manufactured by FLIR (A655sc) and has an accuracy of ± 2°C or ± 2% of the reading. The spectral range, resolution, detector pitch, and NEDT of the camera are 7.5-14 μm, 640x480, 17 μm, and <50 mK, respectively. The high-speed camera was purchased from Photron (Mini-UX-100) and has a pixel resolution of 1280x1024 at 4000 frames per second. During the impact tests, rods attached to the surface through a side wall were used to move the surface after a droplet impact test without opening the chamber. The sodium chloride was purchased from Sigma Aldrich (>99.5% purity).

Contact angle measurements

The superhydrophobic surface used in the droplet impact experiments had a contact angle and standard deviation of 130.8^{o} and 1.3^{o}, respectively. This is based on eight contact angle measurements on four of the tested surfaces (two measurements on each surface).

Data analysis

In the FLIR software, a one-pixel point in the middle of the droplet was chosen to represent the droplet temperature. The software recorded the droplet temperature versus time of each droplet for every experiment. A MATLAB code was written to determine the freezing temperature of each droplet from the data file created in the FLIR software.

Sample fabrication

For the static droplet experiments, Teflon AF1600 was spincoated on the aluminum wafer at 1000 rpm. The sample was then cured on a hot plate at 240 ^{o}C for approximately 10 minutes.

The superhydrophobic surfaces used in the droplet impact experiments were made on copper, purchased from McMaster (99.9% pure). Multiple samples with dimensions of 10.16 cm, 3.81 cm wide, and 3.16 mm thick were made. The top surface of each sample was then roughened first using 80 grit sandpaper followed by 220 grit sandpaper. The samples were then cleaned with soapwater and rinsed with deionized water followed by sonication in acetone for 10 minutes to remove any particles in the crevices. The samples were then dried on a hot plate at 115 ^{o}C for 10 minutes. Teflon AF1600 was then spincoated at 2750 rpm for 30 seconds followed by curing on a hotplate at 260 ^{o}C for approximately 25 minutes.

Surface roughness

The surface roughness of the superhydrophobic surfaces was measured by a profilometer (Detak 6M Stylus). The roughness was measured at three different positions on each Teflon-coated surface. The following table presents the average surface roughness from the three measurements for each sample and the standard deviation.

Table 1. Surface roughness of superhydrophobic surfaces used in droplet impact tests.

	SURFACE ROUGHNESS (nm)	
**Sample**	**Average**	**Std. Dev.**
-10 ^{o}C	1036	118
-20 ^{o}C	1040	164
-20 ^{o}C	925	122
-30 ^{o}C	1460	104
-30 ^{o}C	987	102
-30 ^{o}C	1059	19

-30 ^{o}C	939	152
-40 ^{o}C	942	253
-50 ^{o}C	1005	127

Surface temperature estimation

The surface temperatures reported in the manuscript, i.e., the surface temperature that the droplet contacts, were estimated using a 1-D heat transfer assumption via a thermal resistance-based calculation as:

$$\frac{T_{top} - T_{plate}}{R_{total}} = \frac{T_{top} - T_s}{R_{top}} \quad (1)$$

where T_{top} is the temperature at the top of the droplet as measured with the IR camera, T_{plate} is the plate temperature, which is measured using a 100 Ω platinum RTD with a resolution of 0.01 ^{o}C within the plate, T_s is the desired surface temperature, and R is the thermal resistance, which is defined as the following:

$$R = \frac{L}{kA} \quad (2)$$

where R is the thermal resistance, L is the thickness of the material, k is the thermal conductivity, and A is the cross section area, which is assumed constant in the calculations. The parameter R_{total} is the sum of the thermal resistances of all layers in the calculation. Thermal conductivities of Teflon, water and aluminum are 0.12 W/mK, 0.58 W/mK and 167 W/mK, respectively. Additional details can be found in a previous study by Carpenter and Bahadur[1].

Droplet impact experiments: number of droplets

For the impact experiments, a total of five droplets of each fluid type were tested at -10°C, -40°C, and -50 ^{o}C. Due to the transition temperature occurring between -20 and -30 ^{o}C (and hence, greater scatter in the observed behavior), additional experiments were conducted. At -20 ^{o}C, a total of 10 droplets were tested for each fluid, and at -30 ^{o}C, a total of 20 drops were tested for each fluid.

Thermophysical properties

As described in the discussion section of the manuscript, the following properties were used to estimate the viscous loss and thermal penetration depth. Due to the lack of data on low-temperature properties, the tables below shows data at 4 °C and 20 °C.

Table 2. Thermal properties of pure water and salt solutions at 20 °C and 4 °C.

Temp. (°C)	ρ (kg/m³)		
	Water[2]	Seawater[3]	Brine[3]
20	998	1024	1147
4	1000	1027	1150

Temp. (°C)	μ (mPa s)		
	Water[2]	Seawater[3]	Brine[3]
20	1	1	1.42
4	1.57	1.52	2.13

Temp. (°C)	C_p (J/kgK)		
	Water[4]	Seawater[3]	Brine[3]
20	4183	3999	3424
4	4211	4007	3421

Temp. (°C)	k (W/mK)		
	Water[4]	Seawater[3]	Brine[3]
20	0.59	0.59	0.52
4	0.56	0.58	0.51

Video 1 – droplet impact experiments: This video shows droplets (pure water, seawater, and brine) impacting a superhydrophobic surface at temperatures of -10 ℃, -20 ℃, -30 ℃, -40 ℃, and -50 °C). All videos were recorded at 4000 fps.

Video 2 – stages of freezing: This video shows the initial freezing stage and the second freezing stage of water droplets. The video of the initial freezing stage was recorded at 4000 fps and that of the second freezing stage was recorded at 50 fps.

Video 3 – growth rates during freezing: This video compares the initial freezing stage propagation speed for three droplets (pure water, seawater, and brine). All videos were recorded at 4000 fps.

References

(1) Carpenter, K.; Bahadur, V. Electrofreezing of Water Droplets under Electrowetting Fields. *Langmuir* **2015**, *31*, 2243–2248.

(2) Munson, B.; Young, D.; Okiishi, T. *Fundamentals of Fluid Mechanics*, 5th ed.; John Wiley & Sons, Inc.: Hoboken, NJ, 2006.

(3) Carvalho, G. R.; Chenlo, F.; Moreira, R.; Telis-Romero, J. Physicothermal Properties of Aqueous Sodium Chloride Solutions. *J. Food Process Eng.* **2015**, *38* (3), 234–242.

(4) Mills, A. F. *Heat Transfer*, 2nd ed.; Prentice Hall, Inc.: Upper Saddle River, NJ, 1999.
